# Will Automated Compressing Devices Save More Lives in Recalcitrant Ventricular Fibrillation Cardiac Arrest?

**DOI:** 10.7759/cureus.22407

**Published:** 2022-02-20

**Authors:** Eduardo E Chang, Esther Segura, Sruthi Vellanki, Anup Kumar Trikannad Ashwini Kumar

**Affiliations:** 1 Pulmonary and Critical Care, Union Hospital, Terre Haute, USA; 2 Pulmonary and Critical Care Medicine, Indiana University School of Medicine, Terre Haute, USA; 3 Healthcare Administration, Methodist Health System, Houston, USA; 4 Internal Medicine, Union Hospital, Terre Haute, USA

**Keywords:** cardiac arrhythmia, cardiac arrest outcome, lucas device, ventricular fibrillation (vf) storm, ventricular arrhythmia, pulmonary critical care, in hospital cardiac arrest, chest compressions

## Abstract

We present a 55-year-old male that developed ventricular fibrillation cardiac arrest in the setting of ST-elevation acute myocardial infarction with recalcitrant and persistent ventricular fibrillation arrest that was successfully resuscitated with a good neurological outcome. The persistent chest compressions were performed in our intensive care unit with an automated chest compression system. The patient required defibrillations and nonstop chest compressions which were the key factors for his survival. This is an example we should consider in all our intensive care units. It's time for a paradigm shift in replacing the compressor of a code team with an automated system. The out-of-hospital evidence in acute care is compelling to bring this technology that has been proven crucial in transports from hospital areas, ambulances, helicopters, and ships to the inpatient ICU bedside. In ventricular tachycardia and ventricular fibrillation (Vt/Vf), the electrical storm created is the perfect example of the need to have the best compressions to provide the best care possible with the best survival and neurological outcomes.

## Introduction

Cardiac arrest due to ventricular tachycardia or fibrillation associated with ST-elevation myocardial infarction (STEMI) has a very poor prognosis and mortality. The probability of success generally declines at a rate of 2 to 10 percent per minute of presentation. Early defibrillation and perfusion can make the difference between long-term disability and functional recovery [1-3].

As noted above, refractory ventricular tachycardia or ventricular fibrillation arrest in the setting of STEMI has very high mortality [4,5]. The heart muscle suffers an injury from a ruptured plaque or myocardial infarction which causes further death of heart cells and ventricular fibrillation potentiates further impairment in cardiac output. As the cardiovascular system collapses there is less perfusion to vital organs with the diminished distribution of vital medications and antiarrhythmics [6]. Prompt correction to a perfusion sinus rhythm is key. To do this, consistent uninterrupted chest compressions with prompt defibrillation is necessary. High-quality chest compressions are a key component of proper resuscitation. Studies have shown that coronary perfusion pressure (CPP) >15 mmHg, has the highest possibility of survival. Providing adequate compressions with a rate of 100-120/min and compression depth (2 inches) as well as allowing for full chest recoil while minimizing off time is the cornerstone of cardiac resuscitation [3,7]. Devices that provide mechanical compression of the chest eliminate the shortcomings of human error, lack of consistency, or physical fatigue. The value of automated devices has been recognized in multiple studies on out-of-hospital cardiac resuscitation but the use of in-hospital critical care is not a common occurrence.

## Case presentation

**Figure 1:**
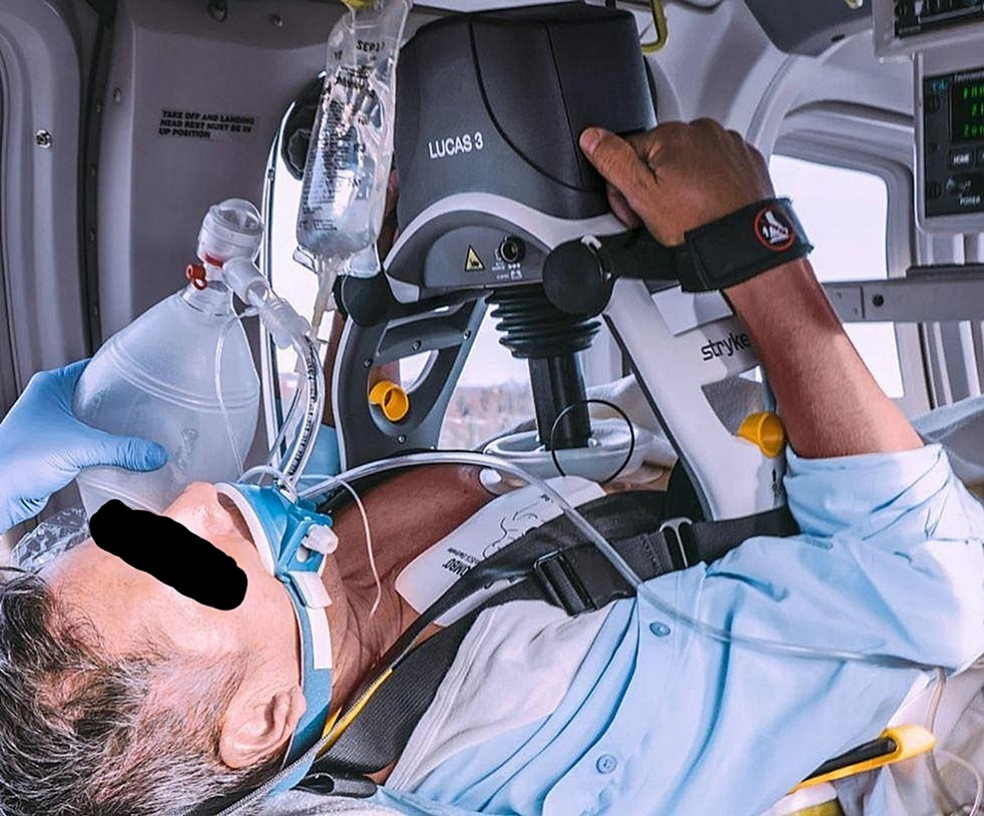
The LUCAS® 3 Chest Compression System

**Figure 2:**
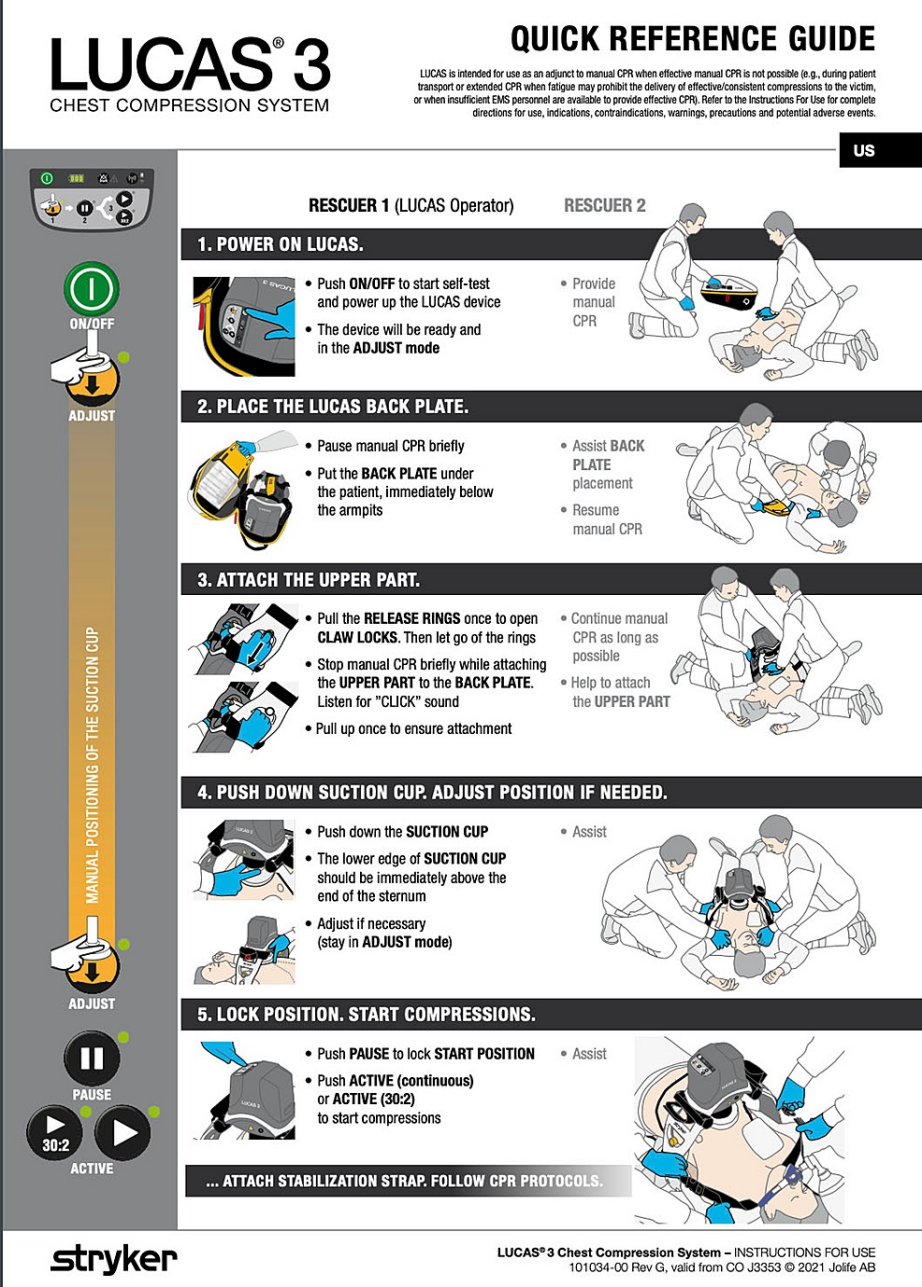
LUCAS Chest Compression System Quick Reference Guide

A 55-year-old male presented to the emergency room, complaining of chest pain that woke him up early in the morning. The patient had substernal chest pain 8/10, received aspirin, oxygen, and morphine. STEMI alert was called, the patient had ST elevation on the inferior leads. The patient was taken to the hemodynamic laboratory (Table 1) and emergent cardiac catheterization was performed. Following placement of the right circumflex stent, the patient was moved to the medical intensive care unit where the patient developed pulseless cardiac arrest secondary to acute anterior wall myocardial infarction. On the rhythm strip, he was noted to be in ventricular fibrillation and prompt advanced cardiac life support (ACLS) protocol was initiated with high-quality cardiopulmonary resuscitation (CPR) and prompt defibrillation was performed early with 200 Joules on a biphasic defibrillator. The LUCAS® Automated Chest Compression System (Jolife AB, a part of Stryker, Lund, Sweden) was placed after the first round of standard CPR (Figures 1, 2).

**Table 1:** Laboratory investigations

WHITE BLOOD CELL COUNT	22x10^3^ /µl
B-TYPE NATRIURETIC PEPTIDE	850 pg/mL
FERRITIN	450 ng/l
D-DIMER	>5 ng/ml

The patient was defibrillated several times as he continued to remain in VT/VF, over 25 minutes. He received several rounds of 1 mg epinephrine every 3-5 minutes, amiodarone IV 300 mg bolus followed by the second dose of IV 150 mg. After 25 minutes of constant CPR performed by the LUCAS® Automated Chest Compression System with multiple shocks, the patient had a Return of Spontaneous Circulation (ROSC), confirmed with end tidal CO2. Within the next 30 minutes, the patient started having purposeful movements and started to wake up. He required fentanyl and a propofol drip for sedation. He remained on vasopressors and amiodarone but the hypothermic protocol was not started due to improvement in mental status. His Arterial Blood Gases (ABG) were 7.23, pCO2 53, pO2 45. The patient also required epinephrine drip at 4 mcg/min and amiodarone. Overnight saturation improved over the course of the next five days, the patient was extubated with discharge to home on day 11 and referred to follow up with cardiac rehab. At the time of discharge, his cerebral performance score was 1 (Good cerebral performance, conscious, alert, able to work, having mild neurological deficit).

## Discussion

Performing cardiopulmonary resuscitation (CPR) can be laborious and fatiguing, the consistency in depth of compressions, quality, and non-interrupted compression is the cornerstone to perfuse the vital organs. CPR can also be an obstacle for percutaneous coronary interventions. Over the past decades, the use of automated electrical devices (AED) delivery of electricity and rhythm detectors has been placed in most public places and the emphasis on quality CPR has been recommended by the American Heart Association. Nevertheless, in most places in America, intensive care units (ICU), emergency rooms (ER), pre-surgical centers, and even some cardiac catheterization laboratories depend on the training and practice of a CPR provider [1,2,4,5]. We have the technology to provide safe, effective, and consistent compressions of the chest during cardiac arrest. The ICU has been the place where pulmonologists and intensivists, support organs such as the lungs during respiratory failure, hemodialysis for kidney failure. The next evolution should be consistent chest compressions during cardiac arrest. In cardiac catheterization, critical care unit, or even in an acute emergency room, the use of the LUCAS®, a rugged device that has proven its efficacy in the outpatient field, could provide constant labor-intensive support. It frees acute care personnel that can provide support to the resuscitating team and maximizes resuscitation efforts [6-8]. The use of LUCAS® Automated Chest Compression System by the critical care physician/nurse in an ICU presents equivalent to manual capabilities in critical care settings, as this frees up manpower for patients to be monitored, supported on a ventilator, measure arterial, venous PaO2 content, and titrate medications. This device can be used in coordination with the catheterization lab to provide ongoing chest compressions up to the point of stent deployment, providing increased resuscitative efforts up to the point of a percutaneous potentially lifesaving intervention.

Chest compressions are the key component of any type of advanced cardiovascular life support and cannot be over-emphasized. The LUCAS® has been used for over a decade in ambulances, on hard concrete, and helicopters. It has proven itself in the acute care setting during high-impact trauma scenarios, subzero hypothermic resuscitation where we don’t let go or call a code until the patient has a normal core temperature which can take hours of resuscitation [2,3]. An automated chest compression system allows rescuers to transport the patient to an intensive care unit for additional lifesaving interventions.

Several animal models have shown that perfusion and mechanical devices can provide good consistent chest compression in transport. Patients that have survived out of hospital cardiac arrest from hypothermia described in case reports where CPR was performed by the automated chest compressors may have not survived using standard one- or two-man CPR. Doing consistent and good-quality CPR for hours in a non-hospitable environment is just not a realistic proposal. Double placebo control trials to show in-hospital use in the ICU setting are lacking but they are difficult to perform. If we extrapolate from the outpatient and paramedic experience, it warrants further consideration. Large metanalyses have suggested that good quality CPR for outpatient resuscitation efforts may be complemented with the compression system.

## Conclusions

The case illustrates the use of an automated system that was helpful to our intensive care team in resuscitating this patient. Good quality CPR for outpatient resuscitation efforts may be complemented with the automated compression system. Acute ventricular fibrillation/tachycardia with no perfusion in the scenario of myocardial infarction presents an opportunity for using the device in a hospital setting. It showed that a mechanical device is able to break the electrical storm by shocking and compressing actively in a nonstop coordinated way, ultimately providing good perfusion, illustrated by ROSC and a PO2 of > 400, 25 minutes after losing the pulse from ventricular fibrillation. The fact that the patient was discharged home with mild neurological sequelae is a testimony for the excellent management of our nurses, respiratory therapist, acute care team, and the use of automated persistent, non-stop consistent compressions, with multiple rounds of defibrillation. The value of automated devices has been recognized in multiple studies on out-of-hospital cardiac resuscitation but the use of in-hospital critical care is not a common occurrence and this needs more case studies and reviews. As pulmonologists, intensive care and acute care specialists we need to consider the next evolution of CPR into an automating and standardizing chest compression system as part of our intensive care units.
